# Sodium cantharidate targets STAT3 and abrogates EGFR inhibitor resistance in osteosarcoma

**DOI:** 10.18632/aging.102193

**Published:** 2019-08-15

**Authors:** Xiang Lu Ji, Ming He

**Affiliations:** 1 Department of Orthopedic Surgery, Shengjing Hospital of China Medical University, Shenyang 110004, People’s Republic of China

**Keywords:** sodium cantharidate, osteosarcoma, STAT3, EGFR, resistance

## Abstract

Osteosarcoma is the most common primary malignant bone tumor in children and adolescents. Overactive EGFR signaling is frequently seen in osteosarcoma cells, and represents a potential therapeutic target. However, feedback activation of STAT3 after EGFR inhibition is linked to treatment resistance, suggesting that combined EGFR/STAT3 inhibition may be needed to overcome this effect. Cantharidin and its analogues have shown strong anticancer effects, including STAT3 inhibition, in several tumor cells. Therefore, we investigated the effects of sodium cantharidate (SC), either as monotherapy and in combination with the EGFR inhibitor erlotinib, on STAT3 activation and osteosarcoma cell growth. Cell viability, migration, and apoptosis assays were performed in human MG63 and U2OS cells, and MG63 xenografts were generated in nude mice to verify the suppression of tumor growth in vivo. Additionally, western blotting and immunohistochemistry were used to verify the STAT3 and EGFR phosphorylation statuses in xenografts. We found that SC repressed cell viability and migration and induced apoptosis in vitro, while combined SC and erlotinib treatment enhanced osteosarcoma growth suppression by preventing feedback activation of STAT3. These data support further development of cantharidin-based combination therapies for metastatic and recurrent/refractory osteosarcoma.

## INTRODUCTION

Osteosarcoma (OS) is the most common primary bone tumor in childhood and adolescence. Current standard therapies include a combination of radical surgery and chemotherapy. Recently, several targeted therapeutic agents have been developed, including inhibitors of phosphoinositide 3-kinase/mammalian target of rapamycin (PI3K/mTOR) [1, 2], tyrosine kinases (TKI) [3], and signal transducer and activator of transcription 3 (STAT3) [4, 5]. However, drug resistance remains a critical obstacle to current pathway-targeted treatments, leading to relapsed or refractory cases and overall poor outcomes.

Multiple drug resistance mechanisms have been illustrated in diverse cancers. For example, the epidermal growth factor receptor (EGFR) T790M mutation impairs the interaction of inhibitory chemicals with the ATP-binding pockets of protein kinases, leading to acquired resistance to first- and second-generation EGFR-TKIs in non-small-cell lung cancer [6]. In lung and breast cancer, MET amplification was shown to mediate resistance to osimertinib (AZD9291) [7, 8]. Furthermore, overactivation of compensatory growth signaling cascades can contribute to cancer cell escape from anti-EGFR therapies. For instance, hyperactivation of STAT3 caused by EGFR inhibitors (STAT3 feedback activation) was shown to mediate resistance in lung cancer [9], colorectal cancer [10] and glioma [11]. Growth factors, e.g. EGF, c-MET, and platelet-derived growth factor receptor (PDGF) activate oncogenic Src, Ras, and members of the Janus kinase (JAK) family, driving phosphorylation of STAT3 in tumors. Phospho-STAT3 forms hetero- or homodimers which translocate to the cell nucleus to promote transcription of pro-survival target genes such as Bcl-2 and matrix metallopeptidase 2 (MMP2). Therefore, preventing STAT3 phosphorylation is an enticing strategy to defeat resistance to EGFR inhibitors such as erlotinib in various tumor types, including relapsed or refractory osteosarcoma.

Cantharidin, a natural toxin produced by the Chinese blister beetle Mylabris, has long been used to treat skin ulcers and warts, and also as an anticancer agent [12]. The broad anti-cancer effects of cantharidin have been studied in different tumor types. A recent report showed that cantharidin induced G2/M phase arrest and reduced osteosarcoma cell viability [13], whereas disruption of the glucose transporter 1/pyruvate kinase M2 glycolytic loop mediated cantharidin inhibition of liver and lung metastases of breast cancer [14]. One of the analogues of cantharidin, sodium cantharidate (SC), promoted apoptosis in hepatocellular carcinoma cells by triggering endoplasmic reticulum stress [15]. Wang et al. found that cantharidin inhibited VEGF-induced JAK1/STAT3 activation and phosphorylation of Akt in human umbilical vascular endothelial cells, leading to suppressed migration and vessel formation [16]. Pan et al., on the other hand, demonstrated that cantharidin downregulated pyruvate kinase M2 (PKM2) and inhibited distant metastasis of breast cancer [14]. Indeed, the PKM2/STAT3 loop has been involved in multiple pathological processes in diverse cancers, influencing proliferation, apoptosis, and angiogenesis [17–19]. The present study tested the hypothesis that cantharidin abrogates feedback STAT3 activation induced by EGFR inhibition in osteosarcoma, resulting in enhanced tumor suppression upon combined SC and erlotinib treatment. While results were promising, further investigations are necessary to confirm the efficacy and safety of SC for osteosarcoma treatment, either as monotherapy or in combination with EGFR signaling inhibitors.

## RESULTS

### STAT3 and EGFR inhibition trigger reciprocal feedback activation in osteosarcoma cells

**Figure 1:**
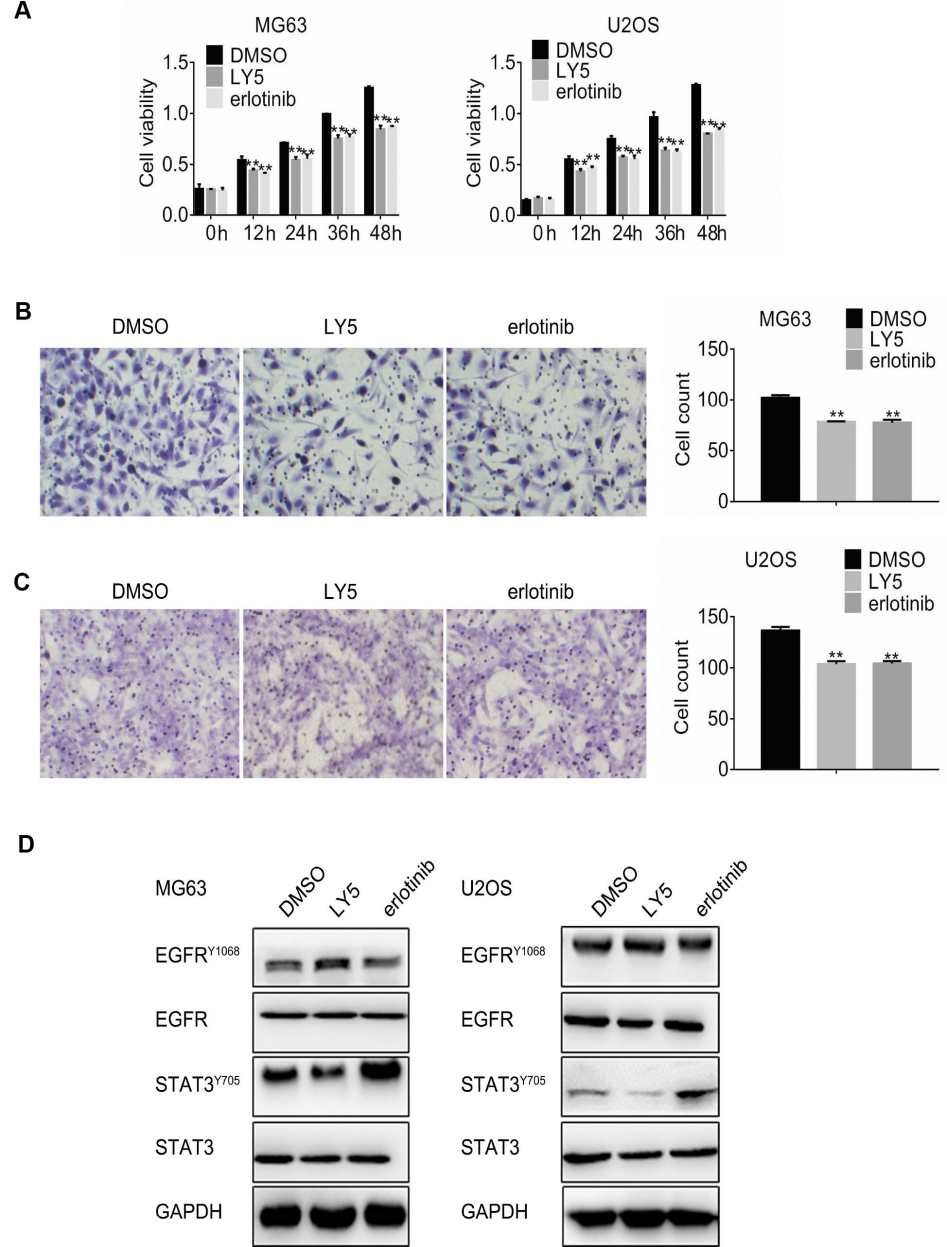
**Independent inhibition of STAT3 and EGFR signaling triggers reciprocal feedback activation in osteosarcoma cells.** (**A**) MG63 and U2OS cells were treated with DMSO (vehicle), LY5, or erlotinib, and the MTT assay was performed to determine cell viability at different time points. Cell migration assay results in MG63 (**B**) and U2OS (**C**) cells treated with DMSO, LY5, or erlotinib for 24 h. Magnification, 200x. Error bars indicate SD. ** p < 0.01 vs. DMSO, n = 3. (**D**). Western blot analysis of phospho-Tyr705 STAT3 and phospho-Tyr1068 EGFR in MG63 and U2OS cells treated with DMSO, LY5, or erlotinib for 24h.

Abnormal EGFR expression and constitutive STAT3 activation have been described in osteosarcoma cells in relation to disease progression and chemotherapy resistance [4, 20]. To determine the effects of STAT3 and EGFR inhibition on osteosarcoma cell proliferation and migration, MG63 and U2OS cells were incubated with the STAT3 inhibitor LY5 (1μM) or the EGFR inhibitor erlotinib (1μM). MTT assay results showed that treatment with either LY5 or erlotinib reduced cell viability significantly compared to control (Figure 1A). Meanwhile, cell migration was also significantly reduced by both treatments (Figure 1B and 1C). Next, western blot was performed to further investigate EGFR and STAT3 phosphorylation status. Both phospho-Tyr705 STAT3 and phospho-Tyr1068 EGFR signals declined after treatment with the respective inhibitors (Figure 1D). However, feedback phosphorylation of EGFR and STAT3 was observed after treatment with LY5 and erlotinib, respectively, whereas no significant differences in EGFR or STAT3 expression were detected post-treatment.

### Sodium cantharidate inhibits osteosarcoma cell growth and migration via STAT3 suppression

**Figure 2:**
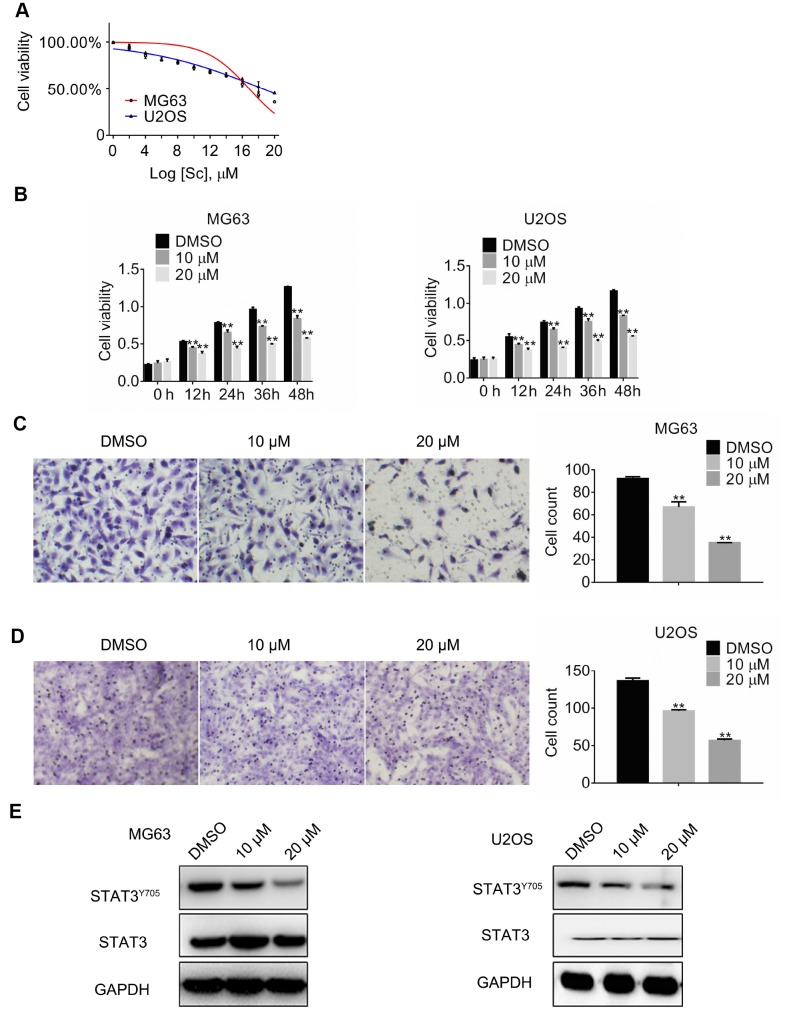
**Sodium cantharidate represses growth, migration, and STAT3 activation in osteosarcoma cells.** (**A**) Half-maximum inhibitory concentration (IC_50_) values obtained for SC. (**B**) MTT assay results in cultured MG63 and U2OS cells exposed to SC. Migration assay results in MG63 (**C**) and U2OS (**D**) cells treated for 24 h with DMSO (vehicle) or SC. Magnification, 200x. Error bars indicate SD. **p < 0.01 vs. DMSO, n = 3. (**E**). Western blot analyses of protein expression in MG63 and U2OS cells exposed over 24 h to DMSO or SC.

Cantharidin, a terpenoid toxin produced by blister beetles, has long been used in traditional Chinese medicine to treat dermatological conditions and as an anticancer agent. To determine its effects on osteosarcoma growth, MG63 and U2OS cells were treated with increasing concentrations of sodium cantharidate (SC; 0–20 μM) for 24 h. Through MTT viability assays, half-maximum inhibitory concentrations (IC_50_) of 16.84 ± 0.28 μM for MG63 cells and 18.5 ± 0.00 μM for U2OS cells were defined (Figure 2A). Therefore, SC at 10 μM and 20 μM was tested in osteosarcoma cells over 0, 12, 24, 36, or 48 h, revealing dose- and time-dependent growth arrest in both cell types examined (Figure 2B). Next, the effect of SC on cell migration was assessed 24 h post-treatment. A dose-dependent reduction in migratory ability was detected in both MG63 and U2OS cells (Figure 2C and 2D).

To investigate whether the observed effects could be related to altered STAT-3 expression, total STAT3 and phospho-STAT3 levels were assessed by western blotting. Results showed that at 24 h post-treatment, SC markedly reduced phospho-Tyr705 STAT3, but minimally affected total STAT-3 expression. These results suggest that suppression of osteosarcoma cell growth by cantharidin may be mediated by a reduction in STAT-3 activation.

### Combined treatment with sodium cantharidate and erlotinib enhances growth arrest and promotes apoptosis in osteosarcoma cells

The effect of STAT3 and EGFR co-inhibition on osteosarcoma cell viability was evaluated by calculating drug combination index (CI) values using the MTT assay. Assayed at 50% effective dose, the LY5/erlotinib combination showed additive anticancer activity (MG63 cells, CI = 1.0097; U2OS cells, CI = 1.0763), whereas the SC/erlotinib combination exhibited synergistic effects (MG63 cells, CI = 0.6340; U2OS cells, CI = 0.6752) (Supplementary Figure 1 and Table 1).

**Table 1:** Combination Index (CI) at ED_50_ values of drug combination on two osteosarcoma cell lines.

**Cell line**	**Drug combination**	**CI at ED_50_**
MG63	LY5 + erlotinib	1.0097
	Sc + erlotinib	0.6340
U2OS	LY5 + erlotinib	1.0763
	Sc + erlotinib	0.6752

Notes: Osteosarcoma cells were treated with various drugs combination as indicated. CI values at 50% effective doses (ED_50_) were calculated using Chou-Talalay method. Data are the mean of two independent experiments performed in triplicate. Sc, sodium cantharidate.

**Figure 3:**
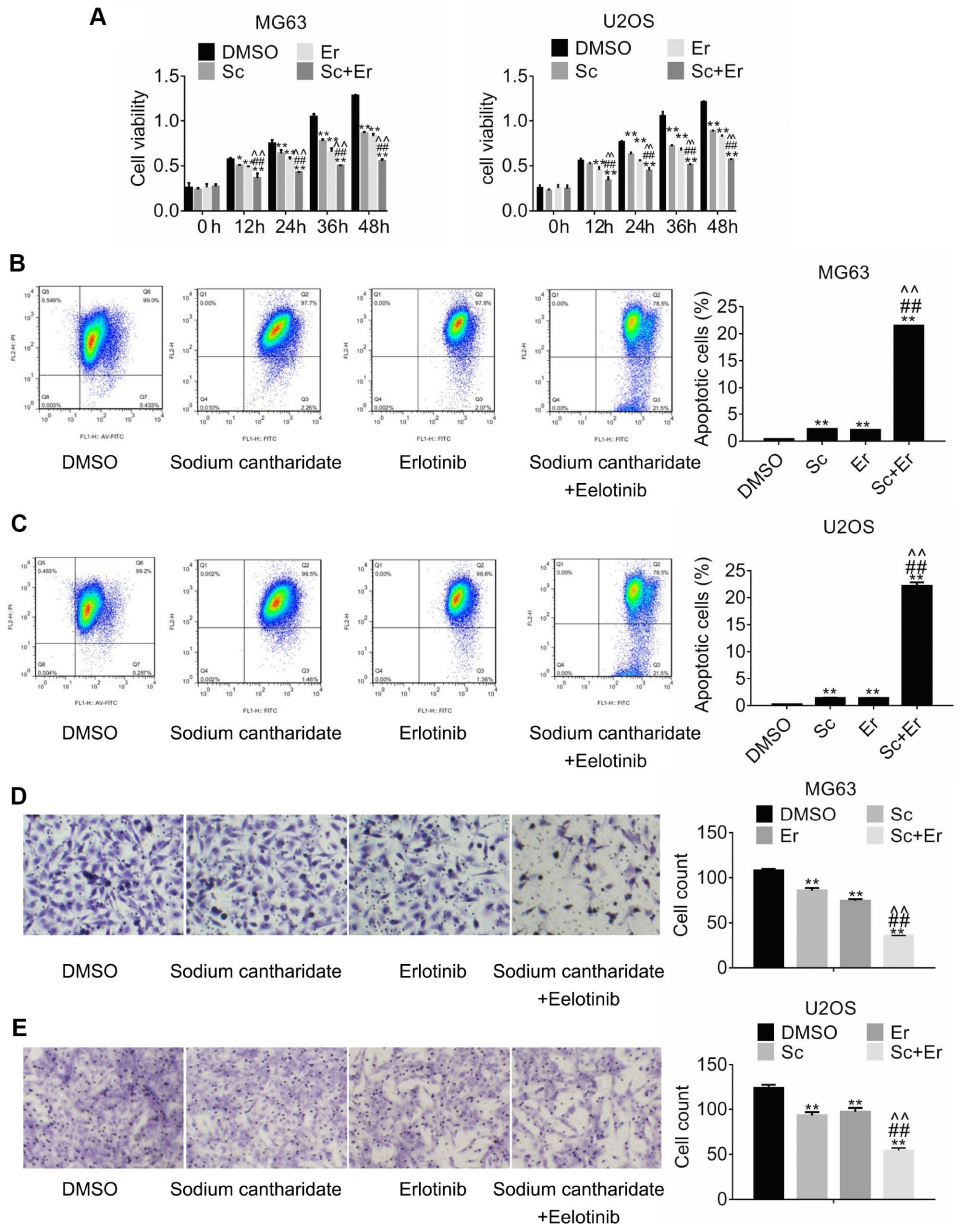
**Combined treatment with sodium cantharidate and erlotinib enhances growth and migration inhibition and promotes apoptosis of osteosarcoma cells.** (**A**). MG63 and U2OS cells were treated with DMSO, SC, erlotinib, or SC plus erlotinib for the indicated times, and the MTT assay was performed to determine cell viability. Apoptosis analysis (JC-1 staining) was conducted in MG63 (**B**) and U2OS (**C**) cells treated as above. Migration assay results in MG63 (**D**) and U2OS (**E**) cells 24 h after individual or combined drug treatment. Magnification, 200x. Error bars indicate SD. **p < 0.01 vs. DMSO. ^##^p < 0.01 vs. SC. ^^p < 0.01 vs. erlotinib; n = 3. Sc, sodium cantharidate; Er, erlotinib.

To explore the potential of combined treatment with SC and erlotinib, MG63 and U2OS cells were incubated with DMSO (control), SC, erlotinib, or a combination of SC and erlotinib for 24 h. Changes in cell viability were measured by MTT assay. As shown in Figure 3A, dual treatment with SC and erlotinib hampered cell growth to a greater extent than either drug alone. To examine the effect of this treatment combination on apoptosis, changes in mitochondrial membrane potential were evaluated through measurements of JC-1 fluorescence by flow cytometry. Consistent with data from cell viability assays, results showed that compared to control, single-drug exposure promoted a significant increase in apoptosis in MG63 cells, while combined treatment further raised the percentage of apoptotic cells (Figure 3B). Identical results were obtained in U2OS cells (Figure 3C). On the other hand, migration assays showed similar effects, namely significant reduction of migration by either drug alone, and compounded inhibition after combination treatment with SC and erlotinib (Figure 3D and 3E).

### Combined exposure to sodium cantharidate and erlotinib suppresses STAT3 and EGFR expression

**Figure 4:**
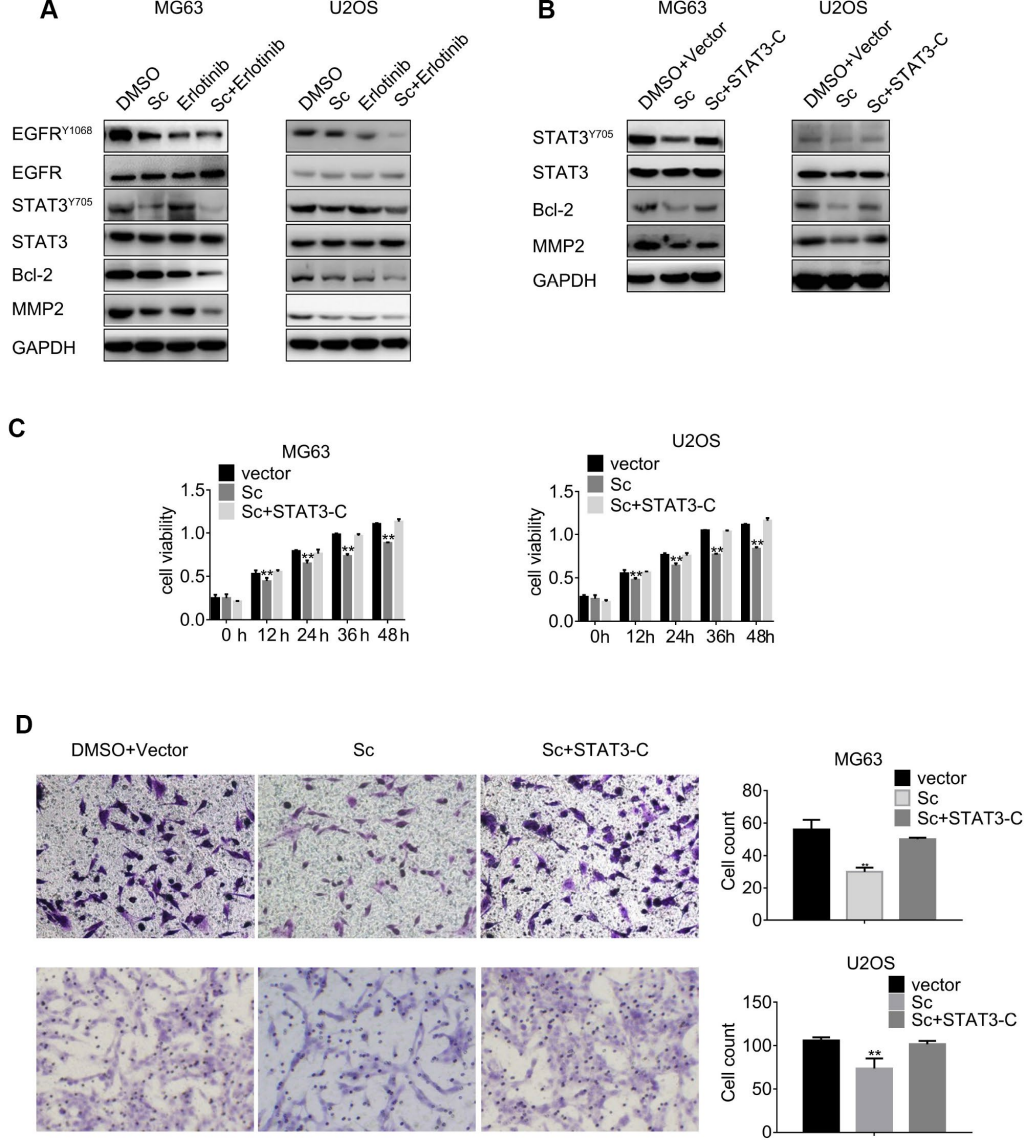
**Combined treatment with sodium cantharidate and erlotinib decreases phosphorylation of STAT3 and EGFR.** (**A**) MG63 and U2OS cells were treated with DMSO, SC, erlotinib, or a combination of SC and erlotinib for 24 h, and phospho-STAT3 and phospho-EGFR expression was evaluated by western blot. (**B**) Western blot analysis of phospho-STAT3, Bcl-2, and MMP2 expression in MG63 and U2OS cells treated with DMSO plus empty vector, SC alone, or a combination of SC and constitutively active STAT3 (STAT3-C) for 24 h. Sc, sodium cantharidate; Er, erlotinib. (**C**) MTT assay results from STAT3-C-transfected MG63 and U2OS cells. (**D**) Transwell migration assay results from MG63 and U2OS cells expressing STAT3-C.

Western blotting was carried out to determine the expression of total and phosphorylated STAT3 and EGFR levels in osteosarcoma cells after combined treatment with SC and erlotinib. As shown in Figure 4A, phospho-STAT3 declined significantly while phospho-EGFR decreased slightly in cells treated with SC. Meanwhile, erlotinib treatment induced feedback activation of STAT3 -denoted by increased phosphorylation-, and this phenomenon was reduced significantly after combined exposure to erlotinib and SC.

Western blotting was next used to investigate the effects of single and combined treatments on the expression of Bcl-2, a pro-survival protein, and matrix metalloproteinase 2 (MMP2), a regulator of extracellular matrix remodelling. Enhanced downregulation of both Bcl-2 and MMP2 was observed after dual drug treatment, compared to the moderate reduction elicited independently by each inhibitor (Figure 4B).

To further assess the link between SC exposure and STAT3 activation status, ectopic expression of constitutively active STAT3 (STAT3-C) was induced in MG63 and U2OS cells. Western blotting showed that STAT3-C rescued STAT3 phosphorylation in the presence of SC, and restored also Bcl-2 and MMP2 expression (Figure 4B). Cell viability and transwell migration assays were next performed in STAT3-C-transfected cells. Results showed that STAT3-C expression abrogated the anti-proliferative and anti-migratory effects of SC (Figure 4C and 4D). These data indicate that SC inhibits STAT3 phosphorylation and can prevent STAT3 feedback activation induced by the EGFR inhibitor erlotinib in osteosarcoma cells.

### Combined treatment with sodium cantharidate and erlotinib inhibits tumor xenograft growth in vivo

**Figure 5:**
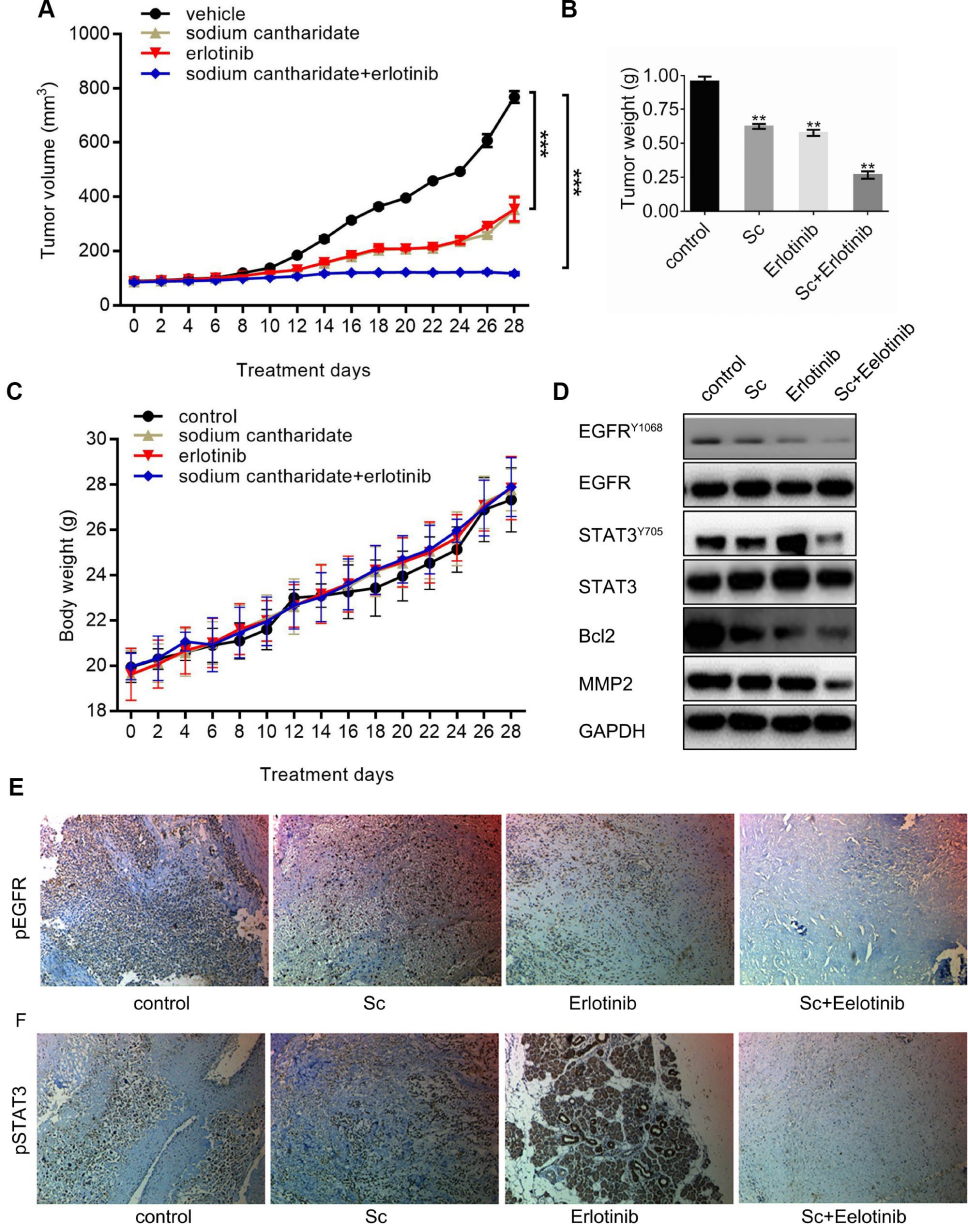
**Combined treatment with sodium cantharidate and erlotinib enhances osteosarcoma growth suppression in nude mice.** Nude mice were subcutaneously implanted with MG63 cells, randomly divided into four groups, and treated with saline (control), SC, erlotinib, or SC plus erlotinib for 4 weeks. (**A**) Mean tumor volumes in each experimental group. (**B**) Tumor wet weights at sacrifice (day 28 post-inoculation). (**C**) Body weight measurements. (**D**) Western blot analysis of phospho-EGFR, phospho-STAT3, Bcl-2, and MMP2 in excised tumor tissues. (**E**) Phospho-EGFR and (**F**) phosphor-STAT3 detection thorough immunohistochemistry in excised xenografts. Magnification, 100x. Sc, sodium cantharidate; Er, erlotinib.

MG63 cell xenografts were generated in nude mice to investigate the effect of dual treatment with SC and erlotinib on tumor growth in vivo. Until sacrifice at day 28 post-implantation, mean tumor volume increased time dependently in mice treated with vehicle (saline), SC alone, erlotinib alone, or their combination (Figure 5A). Xenograft volumes of both SC- and erlotinib-treated mice did not vary from those of vehicle-treated mice until day 12, where they started to show slower growing kinetics. The effect was more pronounced in mice treated with the inhibitors combined, as significantly smaller tumors developed since day 8 post-inoculation (Figure 5A). At sacrifice, mean tumor wet weight in the drug combination group was ~70% lower than control, whereas for single drug treatments a ~40% decrease was recorded (Figure 5B). There were no differences in body weights among treatment groups at the end of the experiment, which suggests good treatment tolerability (Figure 5C). Western blotting was performed to verify the expression of phospho-EGFR and phospho-STAT3 in tumor tissues. As expected, erlotinib-induced STAT3 phosphorylation was reduced notably by SC, while phospho-EGFR levels declined after erlotinib-only and dual combination treatment. Meanwhile, the expression of Bcl-2 and MMP2 was downregulated in parallel with the decrease in phospho-STAT3 (Figure 5D). Lastly, supporting the results of western blots, IHC staining further confirmed reduced activation of EGFR (Figure 5E) and STAT3 (Figure 5F) after combined treatment with SC and erlotinib.

## DISCUSSION

According to the updated statistics of the American Society of Clinical Oncology (ASCO), the general long-term survival rate for localized osteosarcoma is between 60%-80%, while for osteosarcoma with distant metastasis is only 15%-30%. Hence, there is an urgent need for more effective therapies to treat advanced osteosarcomas. Previous studies revealed that EGFR gene copy number amplification and EGFR overexpression are common events in osteosarcoma, so targeting EGFR signaling is a promising therapeutic goal [21, 22]. Activation of EGFR stimulates several downstream signaling cascades, including the RAS/ RAF/MAPK, PI3K/Akt/mTOR, JAK/Src/STAT, and phospholipase C gamma/protein kinase C pathways, triggering oncogene transcription and promoting tumor proliferation, survival, invasion, and drug-resistance [23]. Available therapies targeting EGFR include monoclonal antibodies, e.g. nimotuzumab and cetuximab [24, 25], and tyrosine kinase inhibitors such as erlotinib and gefitinib [26, 27]. However, most current therapies targeting EGFR in osteosarcoma patients have not fulfilled expectations in clinical trials. Increasing evidence points to the STAT3 cascade pathway as a molecular mediator of both intrinsic and acquired resistance to anti-EGFR therapy. Dobi et al. reported that nuclear phospho-STAT3 expression correlated with low objective response rates to cetuximab and chemotherapy in metastatic colorectal cancer [28]. Deeken and colleagues found that persistent, hyperactive STAT3 signaling in advanced solid tumor patients correlated with lack of response to cetuximab treatment [29]. Recently, enhanced STAT3 activation was found in an EGFR-driven, patient-derived xenograft model of non-small cell lung cancer, contributing to acquired EGFR resistance [30].

A large number of chemicals have been tested in the attempt to overwhelm STAT3-mediated drug resistance in tumors. A study showed that inhibition of the EGFR/STAT3 axis by lupeol induced apoptosis in EGFR-TKI-resistant H1975 small lung cancer cells carrying the EGFR L858R/T790M mutation, whereas introduction of a constitutive STAT3 mutant, STAT3-Y705D, prevented this effect [31]. The multi-targeted TKI ponatinib was shown to inhibit STAT3 phosphorylation driven by EGFR and interleukin 6, leading to suppression of colorectal cancer cell growth and migration [32]. Very recently, alantolactone, a natural sesquiterpene lactone, was shown to down-regulate phospho-STAT3 and enhance EGFR inhibition by erlotinib or afatinib in pancreatic cancer [33]. Furthermore, combination strategies were developed to improve anti-tumor effects on osteosarcoma. For example, dual blockage with the pan-HER inhibitor dacomitinib and the STAT3 inhibitor S3I-201 was reported to exert higher growth suppression in sarcoma cells compared to single-drug inhibition [34, 35].

LY5, a small molecule inhibitor that antagonizes constitutive and inducible STAT3 activation, was shown to inhibit tumor cell migration and angiogenesis and to induce apoptosis in medulloblastoma, osteosarcoma, Ewing’s sarcoma, and rhabdomyosarcoma cells [36–39]. In accordance with those studies, we found that LY5 inhibited viability and migration of human osteosarcoma cells. It was reported however that LY5 was ineffective in reducing sarcoma xenograft growth and preventing lung metastasis, therefore more details on the inhibitory effects of LY5, and potentially compensatory tumor responses, need to be obtained [39]. Our evidence for EGFR feedback activation in MG63 and U2OS cells post-LY5 treatment may explain the limited repression of tumor growth upon STAT3 dephosphorylation. Activation of EGFR would bypass the EGFR/JAK/STAT3 axis, initiate other downstream signalling pathways, and compensate for the inactivation of STAT3, favoring osteosarcoma growth and progression.

Research over the last two decades showed that cantharidin and its derivatives can promote tumor regression in multiple cancers via different mechanisms, and numerous analogues were designed to improve efficacy and safety in anti-cancer regimens [12, 40]. Targets of cantharidin include protein phosphatases [41–43], glutathione S-transferases [44], STAT3 [16, 45], Akt [46], and cell division control protein 1 (CDC1) [47]. With focus on the concomitant activation of EGFR and STAT3 as determinants of osteosarcoma progression, we investigated the mechanisms underlying the anticancer effects of SC, a cantharidin analogue, alone and in combination with the EGFR inhibitor erlotinib. Our results showed SC efficacy against osteosarcoma growth and migration in vitro, and prevention of feedback activation of STAT3 induced by erlotinib both in vitro and in vivo. As SC-induced growth suppression was reversed by overexpression of constitutively active STAT3 (where Tyr705 was replaced by an aspartate residue), we conclude that SC anti-tumor effect was phospho-705-STAT3 dependent.

Subcutaneous MG63 xenograft growth inhibition by SC administration in vivo was nearly additive compared to single SC or erlotinib treatment, and was accompanied by declined phospho-EGFR and phospho-STAT3 expression. Interestingly, the inhibition caused by either SC or erlotinib alone diminished by day 26, suggesting onset of acquired resistance. Since mice body weights were not affected, these results implied that the combined therapy was well-tolerated.

**Figure 6:**
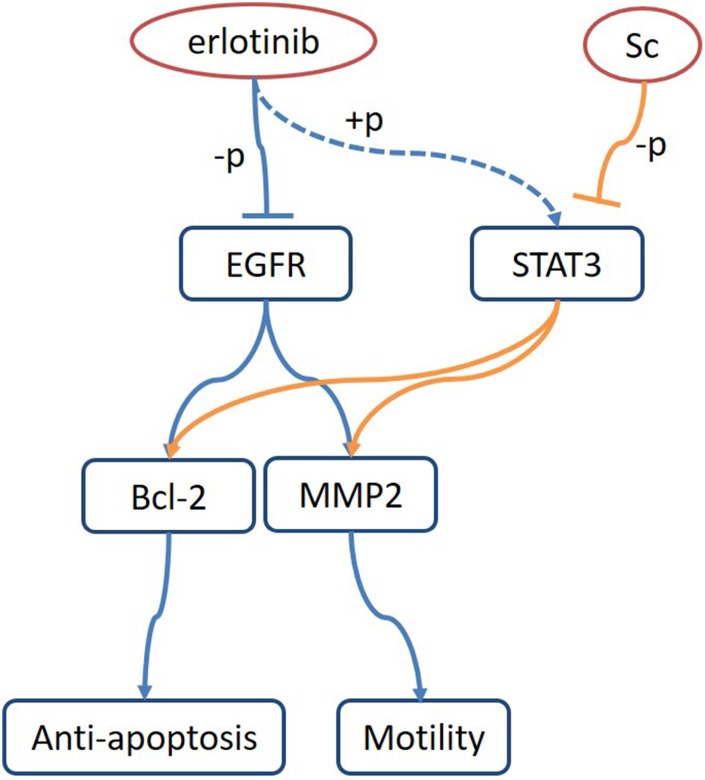
**Proposed model of osteosarcoma growth suppression upon dual treatment with sodium cantharidate and erlotinib.** Combined treatment with sodium cantharidate (Sc) and erlotinib impedes erlotinib-induced activation of STAT3 and signaling through the STAT3/Bcl-2/MMP2 axis, leading to enhanced growth suppression in osteosarcoma.

A complex signaling network underlies feedback activation mechanisms in tumor cells. Ta and colleagues demonstrated that a phosphorylated pro-survival form of the tumor necrosis factor receptor Fas, i.e. Fas.Y291D, enhances EGFR signaling and promotes activation of the nuclear EGF/STAT3 pathway by inducing nuclear accumulation of phospho-EGFR and phospho-STAT3 in colorectal cancer cells. Consequently, proliferation and migration are facilitated by hyperactivation of MEK/ERK and PI3K/Akt signaling [48]. Other studies showed that phospho-STAT3 triggered abnormal dimerization of STAT3, increased the expression of anti-apoptotic proteins Bcl2, Bcl-xl, and MMP2/9, and promoted proliferation, survival, and migration/invasion in ovarian cancer [49, 50], liver cancer [51] and retinoblastoma [52] cells. In line with these findings, our study identified that Bcl-2 and MMP2 expression decreased in parallel with SC-induced STAT3 dephosphorylation both in vitro and in vivo. Figure 6 summarizes the putative mechanisms that underlie STAT3-mediated tumor resistance to EGFR inhibition and reversal of this effect by SC in osteosarcoma. In future investigations, pharmacokinetic/pharmacodynamic analyses along with high-throughput screening of kinase activity are warranted to characterize drug kinetics and identify additional cantharidin targets. Meanwhile, examination of potential mechanisms underlying intrinsic or acquired cantharidin resistance should help define the therapeutic value of SC in cancer treatment.

In conclusion, our data showed that SC, a cantharidin derivative, inhibited osteosarcoma progression in vitro and in vivo and abrogated the feedback activation of STAT3 induced by EGFR inhibition. Our results highlight potential uses of SC in combined therapeutics targeting recurrent and refractory osteosarcoma.

## MATERIALS AND METHODS

### Cell culture and reagents

The human osteosarcoma cell lines MG63 (ATCC^®^ CRL-1427^™^) and U2OS (ATCC^®^ HTB-96^™^) were obtained from American Type Culture Collection (Manassas, VA, USA), and maintained in Dulbecco’s Modified Eagle Medium (DMEM, Sigma–Aldrich, St. Louis, MO, USA). All media were supplemented with 10% fetal bovine serum (FBS, Sigma–Aldrich). These cell lines were employed for the described experiments without further authentication. Sodium cantharidate (CN100506212C) was obtained from Shandong Luoxin Pharmaceutical Co., Ltd (Linyi, Shandong, China). LY5 (#562712) was purchased from MedKoo Biosciences, Inc. (Morrisville, NC, USA). Erlotinib (SML2156) was purchased from Sigma-Aldrich (Shanghai) Trading Co., Ltd (Shanghai, China). The lentivirus expressing a constitutively active mutant form of STAT3 (EF.STAT3C.Ubc.GFP) was a gift from Linzhao Cheng (Addgene plasmid #24983; http://n2t.net/addgene:24983; RRID: Addgene_24983) [53]. Vector pLenti-CMV-MCS-GFP-SV-puro was a gift from Paul Odgren (Addgene plasmid #73582; http://n2t.net/addgene:73582; RRID: Addgene_73582) [54]. Negative control siRNAs (sc-37007) and STAT-3-targeted siRNA (siSTAT3; sc-29493) were obtained from Santa Cruz Biotechnology, Inc. (Shanghai, China).

### Cell viability assay

To evaluate cell viability, 5x10^3^ cells per well were seeded in 96-well plates and cultured for 24 h prior to treatment. After drug treatments, the plates were supplemented with MTT solution at a final concentration of 0.5 mg/mL and further incubated for 4 h at 37°C. 50 μl DMSO was then supplemented after media elimination. The combination index (CI) of LY5 and erlotinib and of sodium cantharidate and erlotinib was calculated and their synergy quantification evaluated using the Chou-Talalay method as described previously [55]. CI < 1, CI = 1, and CI > 1 indicate synergism, additive effect, and antagonism in drug combinations, respectively.

### Migration assay

Migration assays were conducted using modified Boyden chambers with polycarbonate membranes (Nuclepore; Cell Biolabs, Inc., San Diego, CA, USA). Cells (1x10^5^) in 100 μL DMEM supplemented with 0.1% bovine serum albumin were seeded into the upper chambers while the lower chambers were filled with 600 μL DMEM containing 10% FBS. Cells were removed from the upper surface of the filter with a cotton swab post-incubation with test compounds for 24 h. Migrant cells on the lower surface of filter were fixed, stained, photographed and counted under high-power magnification.

### Western blotting

Cell and tissue lysates were resolved by 10% SDS-PAGE and transferred to nitrocellulose membranes. After blocking with 5% skim milk, membranes were incubated overnight with primary antibodies (1:1000), washed thrice with PBS-T, and incubated for 1 h with secondary HRP-conjugated antibodies (1:2500; Cell Signaling Technology, Inc. Shanghai, China). Primary antibodies against phospho-Tyr705 Stat3 (#9145), Stat3 (#9139), phosphor-Tyr1068 EGFR (#3777), EGFR (#4267), Bcl-2 (#15071), MMP2 (#40994), and GAPDH (#5174) were obtained from Cell Signaling Technology, Inc. Shanghai, China. Proteins bands were detected by enhanced chemiluminescence (Pierce; Thermo Fisher Scientific, Inc.).

### Apoptosis assay

The lipophilic dye JC-1 was used to evaluate the loss of mitochondrial transmembrane potential that precedes apoptosis. Cultured MG63 and U2OS cells (2.5x10^5^) were collected 24 h post-treatment, washed once with 1X dilution buffer, and stained with 1 μM JC-1 for 30 min. Flow cytometric analysis was performed to evaluate JC-1 fluorescence, according to instructions in the BD^™^ MitoScreen (JC-1) Kit (#551302; BD Biosciences, San Jose, CA, USA).

### Tumor xenografts

Animal experiments were approved by the Institutional Animal Care and Use Committee of Shengjing Hospital of China Medical University. A total of 5×10^6^ MG63 cells in 0.2 mL of media were subcutaneously injected into athymic nude male mice. Tumor-bearing mice were randomized into four groups (n = 3-4 mice per group) when mean tumor volume reached approximately 100 mm^3^. Each group received either oral saline, i.p. sodium cantharidate (10 mg/kg/d), oral erlotinib (50 mg/kg/d), or dual treatment with sodium cantharidate (1mg/kg/d), and erlotinib (50 mg/kg/d). Tumor volume and body weight were measured every other day. At the end of the studies, all animals were euthanized humanely by cervical dislocation under isoflurane anaesthesia.

### Immunohistochemistry

Tumors were excised, fixed in formalin, embedded in paraffin and sectioned. Representative sections were analyzed using the IHC staining kit Ultra-Sensitive^TM^ SAP (KIT-7710, Maixin. Biological, Fuzhou, China) after incubation with either phosphor-Tyr1068 EGFR (#3777) or phospho-Tyr705 Stat3 (#9145) antibodies (Cell Signaling Technology, Inc. Shanghai, China.).

### Statistics

Statistical analyses were performed using GraphPad Prism 7 software (La Jolla, CA, USA). Data from at least three independent experiments are presented as the mean ± standard deviation (SD). Group means differences were analyzed by Dunnett’s multiple comparisons test. P < 0.05 was considered statistically significant.

## Supplementary Material

Supplementary Figure 1
