# Supplementary material for: Sodium cantharidate targets STAT3 and abrogates EGFR inhibitor resistance in osteosarcoma
Source: Aging (Albany NY). 2019 Aug 15;11(15):5848–63. doi: 10.18632/aging.102193 (PMC6710037; doi:10.18632/aging.102193)
Supplement: Supplementary Figure 1 [file aging-11-102193-s001.pdf]

SUPPLEMENTARY FIGURE

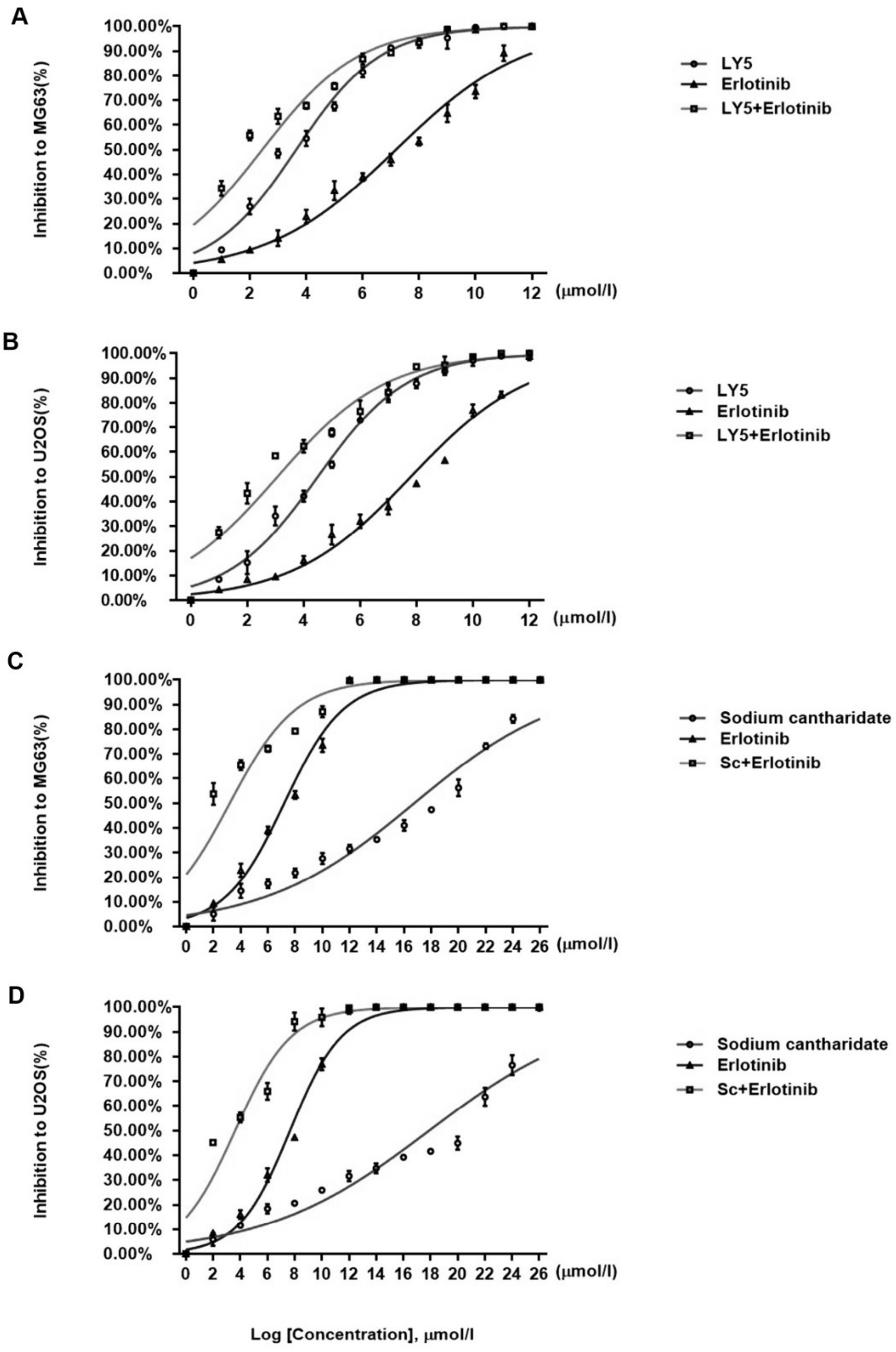

Supplementary Figure 1. Drug combination index (CI) estimations. (**A**) CI for LY5 and erlotinib in MG63 cells. (**B**) CI for LY5 and erlotinib in U2OS cells. (**C**) CI for sodium cantharidate and erlotinib in MG63 cells. (**D**) CI for sodium cantharidate and erlotinib in U2OS cells.
